# Mechanistically transparent models for predicting aqueous solubility of rigid, slightly flexible, and very flexible drugs (MW<2000) Accuracy near that of random forest regression

**DOI:** 10.5599/admet.1879

**Published:** 2023-08-21

**Authors:** Alex Avdeef

**Affiliations:** in-ADME Research, New York, NY 10128 USA

**Keywords:** General solubility equation (GSE), Abraham solvation equation (ABSOLV), flexible-acceptor GSE(*Φ,B*), Consensus model, decision-tree Exclusive Or (XOR) model, Kier molecular flexibility index (*Φ*), drug-like molecules, machine learning (ML), intrinsic solubility

## Abstract

Yalkowsky’s General Solubility Equation (GSE), with its three fixed constants, is popular and easy to apply, but is not very accurate for polar, zwitterionic, or flexible molecules. This review examines the findings of a series of studies, where we have sought to come up with a better prediction model, by comparing the performances of the GSE to Abraham’s Solvation Equation (ABSOLV), and Random Forest regression (RFR) machine-learning (ML) method. Large, well-curated aqueous intrinsic solubility databases are available. However, drugs may be sparsely distributed in chemical space, concentrated in clusters. Even a large database might overlook some regions. Test compounds from under-represented portions of space may be poorly predicted, as might be the case with the ‘loose’ set of 32 drugs in the Second Solubility Challenge (2020). There appears to be still a need for better coverage of drug space. Increasingly, current trends in predictions of solubility use calculated input descriptors, which may be an advantage for exploring properties of molecules yet to be synthesized. The risk may be that overall prediction approaches might be based on accumulated uncertainty. The increasing use of ML/AI methods can lead to accurate predictions, but such predictions may not readily suggest the strategies to pursue in selecting yet-to-be-synthesized compounds. Based on our latest findings, we recommend predictions based on both ‘grouped’ ABSOLV(GRP) and ‘Flexible Acceptor’ GSE(*Φ*,*B*) models with the provided best-fit parameters, where *Φ* is the Kier molecular flexibility index and *B* is the Abraham H-bond acceptor strength. For molecules with *Φ* < 11, the prudent choice is to pick the Consensus Model, the average of ABSOLV(GRP) and GSE(Φ,B). For more flexible molecules, GSE(Φ,B) is recommended.

## Introduction

The pursuit of accurate prediction of solubility of drugs from molecular structure is still evolving and continues to be challenging [1-7]. It had been proposed that shortfalls have been due to the lack of high-quality solubility data from the chemical space of drugs. But there has been some pushback to that view [6]. Since 2011, we have been actively collating and harmonizing published values of pH-dependent aqueous solubility of drug-like and drug-relevant molecules of importance in the discovery-to-development stages of pharmaceutical research, aggregated in the Wiki-p*S*_0_™ database (*in-ADME* Research). A book tentatively entitled “Predicting Solubility of New Drugs - Handbook of Critically Curated Data for Pharmaceutical Research” is under review by a publisher [7]. It tabulates solubility data up to 2022. The collection comprises 3695 different substances, with 7619 entries of intrinsic solubility (uncharged form). Considerable effort has been put into deciding that the data are of high quality, based on carefully selected published sources, guided in part by the best-practices ‘white papers’ recently published [8-12]. Just about all entries in the database are referenced to primary sources.

The reproducibility of statistical methods to predict solubility at best has hovered around the root mean square error (RMSE) of 0.6 log unit but is typically RMSE > 1 in many studies [1-5]. On the other hand, it has been firmly estimated that the average interlaboratory reproducibility can be as low as 0.18 log unit in carefully curated databases, which includes correcting reported solubility for ionization (*i.e*. deriving intrinsic solubility, *S*_0_) and by normalizing for temperature (by transforming measurements performed in the range 10-50 to 25 °C) [7,13-20]. Consistent solubility unit conversions, methods of phase separation, and procedures for measuring *pH* also play critical underpinnings in data quality [8-12].

This review discusses a recent series of five interrelated publications [16-20], where the use of several computational methods to predict intrinsic solubility were explored: (a) Yalkowsky General Solubility Equation (GSE) [21-26], (b) Abraham solvation equation (ABSOLV) [27-29], (c) Avdeef-Kansy Flexible-Acceptor General Solubility Equation (a.k.a., GSE(*Φ,B*)) [18], (d) Consensus of ABSOLV and GSE(*Φ,B*) [19], and (e) Breiman Random Forest regression (RFR) statistical machine learning (ML) method [30-33]. The above data-driven methods were trained with the Wiki-p*S_0_* database. The traditional GSE is often considered pre-trained. It is popular for its simplicity and ease of use. New prediction methods are often benchmarked against the GSE.

This review concludes with the introduction of a new variant method induced from the above five studies, called the Exclusive Or (‘XOR’) Decision Tree model, drawing on ABSOLV and/or GSE(*Φ,B*) models, depending on the value of *Φ.* The method may be useful when a large diverse database of intrinsic solubility values of drug-like or drug-relevant molecules is available. It mirrors the Consensus model [19].

## Analytic continuity of methods to predict solubility of drug-like molecules

The solubility (intrinsic, log molarity units) calculated by GSE depends on the value of the octanol-water partition coefficient (measured log *P* or calculated clog *P*) and the measured (or calculated) melting point (*mp* / °C) of the molecule. No further training is required for this thermodynamically well-founded legacy equation.



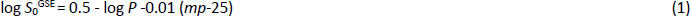



The Abraham and Le [28] ABSOLV equation to predict solubility takes the form:



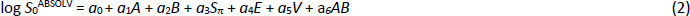



In the multiple linear regression (MLR) equation, the log *S*_0_ is the dependent variable (measured log intrinsic molar solubility) and the independent variables are the five solute solvation descriptors accounting for the energy of transfer of solute from solid to the solution phase: *A* is the sum of H-bond acidity (donor potential), *B* is the sum of H-bond basicity (acceptor potential), *S_π_* is the dipolarity/polarizability (subscripted here, so as not to be confused with solubility), *E* is an excess molar refractivity in units of (cm^3^·mol^-1^)/10, and *V* is the McGowan characteristic molar volume in units of (cm^3^·mol^-1^)/100. The *a*_0_ - *a*_6_ constants in Eq. (2) are determined by regression based on the training database of intrinsic solubility values. The five solvation descriptors may be calculated from 2D structure (provided in SMILES notation or as coordinates in a ‘mol’ file) using the program ABSOLV [29] (*cf*. www.acdlabs.com).

As detailed elsewhere [18-20], the Flexible-Acceptor model, GSE(*Φ,B*), was developed as a critical expansion of the legacy GSE, following an exhaustive search of descriptors in a principal components analysis. For the original three constants (0.5, -1.0, -0.01) in Eq. (1), the first two were fitted to an exponential function of the sum of two descriptors, *Φ* (Kier’s [34] molecular flexibility index) and *B* (Abraham’s [27-29] H-bond acceptor potential). The third crystal-lattice contribution term was best characterized as a linear function of *Φ+B*.



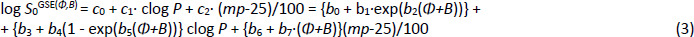



The molecular flexibility descriptor, *Φ*, has been defined [34] in terms of structural attributes (chains, rings, branches, atom counts) as *Φ* = ^*1*^κ· *^2^κ* /NHA, where ^*1*^κ and *^2^κ* are the first- and second-order topological shape indices, and NHA is the heavy atom count in the molecule (RDKit descriptors from http://www.rdkit.org). In the Wiki-p*S*_0_ database, *Φ* values ranged from 0.4 to 43. For molecules with molecular weight > 500 Da, the 630 *Φ* values range from 4.9 to 43, averaging 12. *B* (*cf*. Eq. 2) for the large molecules ranges from 0.4 to 12.9, averaging 3.6. In the development of the SGE(*Φ*,*B*) model, several combinations of *Φ* and single Abraham descriptors were examined. The sum of *Φ* and *B* improved performance over just *Φ* in Eq. (3) [18-20].

The eight b_0_-b_7_ constants in the GSE(*Φ,B*) model were determined in two steps. (i) The log *S*_0_ values in the training set were sorted on *Φ+B*. The sorted data were then divided into 20 bins, each of near-equal values of *Φ+B*. Seven of the lowest-(*Φ+B*) bins contained an average of nearly 1000 log *S*_0_ values per bin. The data in each bin were then analyzed to find the best-fit c_0_, c_1_ and c_2_ constants in the first line of Eq. (3), using PLS regression. (ii) The resultant 20 *c*_0_, *c*_1_, and *c*_2_ constants from the bins were then displayed in three plots against (*Φ,B*) values, to suggest possible nonlinear fitting equations. From this, the analytical expressions for the c-coefficients were determined by standard nonlinear least-squares methods, as detailed elsewhere [18-20].

Of the new ML statistical approaches, the RFR method is thought to be among the premier performers in prediction accuracy, although deep-learning neural network methods may be as good [3,4]. The RFR software is freely downloadable and is easy to use (*cf*. ’random forest’ library for the R statistical software [31-33]). The method works by constructing an ensemble of hundreds of decision trees based on a random selection of a portion of the training set of solubility measurements, using hundreds of randomly-grouped descriptors provided by the user. It is not possible to state a simple explicit equation, like Eqs. (1)-(3), for the RFR method.

In this study, all metrics expressed as the coefficient of determinations (*r^2^*) and root-mean square errors (RMSE) are of the ‘validation type’ [35], unless otherwise indicated (see Abbreviations and definitions). Also, all logarithm functions are with reference to base 10.

### Random forest regression trained with the Wiki-pS_0_ Database

In the first of our recent solubility prediction studies [16], four test sets were examined:

- **Test Set 1** – Yalkowsky-Banerjee [22] set of 21 organic nonelectrolytes (6 solid and 3 liquid poorly-soluble pesticides, 11 older drugs, and a dye/laxative molecule), with the test set mean log *S*_0_ = -3.85 (log molarity). This set has been widely tested by other investigators, to assess the effectiveness of prediction models.
- **Test Set 2** – Hopfinger *et al*. [2] set of 28 well-studied drugs (all ionizable), with mean log *S*_0_ = -4.03. This test was part of the first solubility challenge [1,2].
- **Test Set 3** – Llinas-Avdeef [3] high-consensus ‘tight’ set of 100 drugs, with the mean log *S*_0_ = -4.03. This test was part of the second solubility challenge [3,4].
- **Test Set 4** – Llinas-Avdeef [3] low-consensus ‘loose set’ set 32 drugs, with the mean log *S*_0_ = -5.24. This test was also used in the second solubility challenge [3,4].

It was found that *all* the predictions generated negative bias (-0.08 to -0.65), in many cases suggesting that solubility of low-soluble compounds was *over*estimated. RFR outperformed the GSE and ABSOLV methods on all common metrics [35], averaged over the four test sets: *r*^2^_avg_ = (0.69, 0.47, 0.42), RMSE_avg_ = (0.92, 1.17, 1.17), MPP_avg_ (measure of prediction performance [2]) = (51, 38 and 32 %), for RFR, ABSOLV and GSE, respectively. It is interesting that the RFR method predicted Yalkowsky-Banerjee Set 1 only marginally well: *r*^2^ = 0.82, RMSE = 0.83, MPP = 57 %, bias = -0.23. When 115 practically-insoluble agrochemicals were added to the Wiki-p*S*_0_ database (which had been devoid of such pesticides), the performance improved: *r*^2^ = 0.89, RMSE = 0.63, MPP = 71 %, bias = +0.02. This confirmed the importance of matching the chemical space of test molecules with those in the training set. However, the ability to predict the solubility of drugs to the level of the quality of measured data remained out of reach (*RMSE* 0.6 *vs.* 0.2). Improvements in the methods (*e.g*. more effective descriptors) [6] and better coverage of the clustered [36] chemical space of drugs were called for.

The principal component analysis (PCA) based on the 30-most important descriptors identified in RFR yielded a scores plot of the first two principal components for the training set solubility, showing a very dense symmetrical distribution about the origin for molecules with molecular weights (MW<500 Da). Large molecules (MW>800 Da) were sparsely (but near diagonally) represented in the bottom-right quadrant, giving the overall distribution a ‘comet-like’ head-tail appearance [16]. Small molecules in the Lipinski [36] Rule of 5 (Ro5) chemical space, populating the ‘head,’ thus appeared to have very different distributions than those beyond the Ro5 (bRo5) space in the ‘tail.’

### Small Ro5 molecules can predict the solubility of large bRo5 molecules, using the RFR method

In our second study [17], the above ‘comet-like’ PCA distribution enticed us to explore whether small molecules (Ro5 space) in a training set could be used to predict the solubility of large molecules (bRo5 space), although at first, we were not optimistic of a good outcome. The molecules with MW>800 Da were selected as the test set, with the rest of the database molecules serving as the training set.

- **Test Set 5** – Avdeef-Kansy [17] - test set of 31 ‘big’ drug-like/drug molecules with MW 802-1882 Da (*e.g*. cyclosporine A, gramicidin A, leuprolide, nafarelin, oxytocin, vancomycin), with mean log *S*_0_ = -4.52.

It was found the RFR *method spectacularly* distinguished itself from the simpler GSE and ABSOLV models: *r*^2^ = (0.37, -5.24, -3.82), RMSE = (1.07, 3.36, 2.95), MPP = (42, 10 and 16 %), *bias* = (+0.30, +2.64, +0.16), for RFR, ABSOLV and GSE, respectively. In probing this further, the first two normally-fixed parameters in the GSE(classic) model (+0.5 and -1.0) were ‘re-trained’, to yield log *S*_0_^GSE^_SMALL_ = -0.28-0.83 clog*P* -0.01·(*mp* - 25). The modified versions of the Yalkowsky’s equation used as the training set produced metrics comparable to those of the RFR method: *r*^2^ = 0.33, RMSE = 1.10, MPP = 30 %, and bias = +0.04. A similar treatment for the large molecules yielded log *S*_0_^GSE^_BIG_ = -1.77-0.40 clog*P* -0.01·(*mp* - 25). These modifications showed that c_0_ and c_1_ could be variable coefficients, rather than constants. It was this observation that pointed the way to the development of the Flexible-Acceptor model, GSE(*Φ,B*), which turned out to be quite an improvement over the classic equation, providing realistic coverage of the chemical space of drugs up to MW of about 2000 Da.

### Flexible-acceptor GSE(Φ,B) for predicting the solubility of large molecules

When it became evident that the GSE might be beneficially re-trained to predict a broader class of compounds, especially large drug-like molecules, a search was made for analytical functions which could represent the three traditional constants in terms of meaningful property descriptors, guided by the PCA distribution mentioned above [16]. The novel Flexible-Acceptor model (*cf*. Eq. (3)), GSE(*Φ,B*), was the outcome of the effort [18].

As the last section indicated, it had been a challenge to predict solubility of large molecules using simple but easily transparent and interpretable models such as GSE and ABSOLV (as indicated by *r*^2^ < 0 and RMSE ≥ 3). RFR showed promise, but this ML method can be opaque, given that over 200 calculated descriptors are often used in the random mixing of portions of the learning set with randomly-selected subsets of descriptors. Nevertheless, there are good reasons to attempt the prediction of the solubility of large molecules. Many drugs (mostly derived from natural products) in immunosuppression, oncology, and for the treatment of infectious/viral diseases are large, lipophilic, and possess many H-bond acceptors. Literature discussions highlight the promise of therapeutic opportunities for ‘beyond the Rule of 5’ (bRo5) molecules [37-44]. Caron and colleagues [42-44] have paved the way for recognizing the importance of Kier’s molecular flexibility index, *Φ*, in predicting the physical properties of bRo5 molecules.

Flexible molecules with the potential to form intramolecular H-bonds may possess enhanced solubility in polar media (*i.e*. water), by adopting hydrophilic ‘extended’ conformations, as well as enhanced permeability across apolar cell membranes, by adopting hydrophobic ‘folded’ conformations [37-44]. Given that large molecules may pose pharmacokinetic (PK) risks due to low solubility, the need for caution is especially important. So, reliable, and actionable *in silico* models to predict solubility before such molecules are prioritized for synthesis could be a valuable contribution in PK risk assessment.

- **Test Set 6** – Avdeef-Kansy [18] – One additional molecule was added to Set 5 to comprise Test Set 6 of 32 drugs with MW from 802 to 1882 Da, MW_avg_ = 1037 Da. Average log *S*_0_ = -4.61 (range -1.2 to -7.6); clog*P*_avg_ = 3.3 (-3.6 to +17.9); *B*_avg_ = 5.8 (1.9-11.6); *Φ*_avg_ = 20 (11-41).

Using all the database molecules with MW < 800 Da as the training set, the GSE(*Φ,B*) prediction of larger molecules yielded promising statistics: *r*^2^ = 0.40, RMSE = 1.10, MPP = 41 %, bias = -0.08. By contrast, the traditional GSE generated *r*^2^ < 0, RMSE = 3.0, and MPP = 16 %. RFR, the ‘gold standard’ of accuracy in the minds of some computational chemists, generated *r*^2^ = 0.37, RMSE = 1.07, MPP = 38 %, bias = +0.30. Overall, RFR and GSE(*Φ,B*) performances were about the same. This encouraging result became the segway to our study of a more diverse class of test molecules: recently FDA-approved drugs (2016-2022).

GSE(*Φ*,*B*) works well for both big and small molecules, but the Consensus model, based on the average of GSE(*Φ*,*B*) and ABSOLV(GRP) can be even slightly better than the RFR Model

The pharma R&D productivity trended downwards from a high point in 1996 to leveling off by 2010, judging by the count of new molecular entities (NMEs) approved each year [45]. From 2011 to 2020, an upward recovery trend, albeit bumpy, can be discerned. More recently, a trend reversal may be taking place. Of the drugs approved in 2020 and 2021, 72 % are considered ‘small molecule’ NMEs. But even these are getting larger, less soluble, more lipophilic, and possessing more H-bond acceptors, when compared to older drugs. Large molecules may be burdened with PK risks, as noted above.

In our fourth [19] and fifth [20] studies, we directed our efforts to predict the solubility of newly-approved drugs, covering the period of 2016-2021. A few newly-approved drugs were added from 2022 [7]. It was of particular interest to see how the Flexible-Acceptor model, GSE(*Φ,B*), would perform, since many of the new drugs are large and draw on a diverse chemical space. In addition to predictions, the trends in physicochemical properties of these new drugs were quantitated [20] – property inflation was evident. To reduce method bias in ABSOLV prediction, the training set data were divided into six groups. The new operational variant [20], called ABSOLV(GRP), is described below.

- **Test Set 7** – The intrinsic solubility of 105 newly FDA-approved drugs (2016 to 2022) were added to the *Wiki-pS_0_* database [7]. Average log *S_0_* = -4.64 (ranging -8.5 to +0.6); *MW_avg_* = 465 Da (174-1215), *clogP_avg_* = 3.3 (-5.8 to +8.7); *B_avg_* = 2.2 (0.8-7.7); *Φ_avg_* = 6.7 (1.9-32.0).

The GSE(*Φ,B*) was originally developed to predict the solubility of large flexible drug-like molecules. It was shown to predict the solubility of drugs beyond Lipinski’s ‘Rule of 5’ chemical space (bRo5) to a precision matching that of the Random Forest regression (RFR) machine learning method [18]. Surprisingly, the GSE(*Φ,B*) appeared to work well *also* for Ro5 drugs [19]. As before, to add context to the GSE(*Φ,B*) model, GSE(classic), ABSOLV(GRP), and RFR models were also applied to predict log *S*_0_ of the newly-approve ‘small molecule’ NMEs, for which useable reported solubility values could be accessed (the majority from FDA New Drug Application published reports). The prediction models were retrained with an enlarged version of the *Wiki-pS_0_* database. GSE(classic) was applied in its traditional form.

RFR and GSE(*Φ,B*) outperformed the GSE(classic) and ABSOLV(GRP) models in most of the metrics [35]: *r*^2^ = (0.64, 0.59, 0.47, 0.41), RMSE = (1.09, 1.15, 1.33, 1.40), MPP = (37, 32, 29 and 31 %), and bias = (-0.11, -0.34, +0.01, -0.33) for RFR, GSE(*Φ,B*), ABSOLV(GRP), and GSE(classic), respectively, for the 105 new drugs. The Consensus model [19] (average of GSE(*Φ,B*) and ABSOLV(GRP)), performed just about as well as the RFR model, with the metrics: *r*^2^ = 0.63, RMSE = 1.10, MPP = 34 %, and bias = -0.16.

The near zero bias of the ABSOLV(GRP) model in the most recent studies was largely achieved by dividing the training set into six sub-classes. Big molecules (MW > 800 Da) and quaternary ammonium compounds were first removed from the training set and were each analyzed separately, with the a_0_-a_7_ coefficients in Eq. (2) determined by PLS regression. The remaining training set molecules were divided into four classes, based on their net charge at pH 7.4 : acids (-), bases (+), zwitterions (±), and neutrals (0). Table 1 summarizes the results of the sub-class training.

The GSE(*Φ,B*) model was further re-trained, as more data had been added to the *Wiki-pS_0_* database. For this step, the solubility data in the training set were sorted on *Φ*+*B* and grouped into twenty bins of increasing values [19]. In each *Φ*+*B* bin of about 700 solubility entries, the three GSE coefficients, *c*_0_-*c*_2_ in Eq. (3) were each determined by linear PLS regression. The resultant three groups of c-coefficients showed recognizable forms as a function of *Φ*+*B*. The trend in *c*_0_ was characteristic of a decreasing exponential function in *Φ*+*B*, suggesting that the solubility of a liquid solute in model lipid (octanol) [16-18] decreases as *Φ*+*B* increases. The trend in the *c*_1_ coefficients (lipophilicity factors) was that of an increasing exponential function, indicating a decreasing influence due to lipophilicity as *Φ*+*B* increases. The slightly increasing *c*_2_ coefficients (crystal lattice effect) could be fit to an ascending linear form as a function of *Φ*+*B.* Apparently, crystal lattice contributions are not appreciably altered by molecular flexibility and H-bond acceptor character, and trend near the traditional value (-0.01) in Eq. (1). Evidently, solubility dependence on flexibility and H-bond acceptor strength are mediated by solution-phase interactions [46]. Table 2 summarizes the most-recently trained GSE(*Φ,B*) parameters.

The Consensus prediction equation is simple in form and can be easily incorporated into spreadsheet calculations (using the parameters in Tables 1 and 2), which is not the case for the RFR model.

Aside from opaqueness, there are other limitations to RFR. Since the prediction is the average log *S_0_* value of several training set molecules with descriptors most like those of the test compound, the RFR model, as it is currently implemented, cannot extrapolate beyond its training space. So, for molecules much less soluble than those in the training set, the prediction always overestimates the solubility. Consequently, if a test molecule is also inadvertently included in the training set, RFR will very likely present the experimental value as the prediction.

## Consensus *vs.* Exclusive Or (XOR) models

In prediction of the solubility of newly-approved drugs [19,20], the RFR and GSE(*Φ,B*) models outperformed the GSE(classic) and ABSOLV(GRP) models in most of the metrics, as noted above. As a bonus, the Consensus model based on the average of GSE(*Φ,B*) and ABSOLV(GRP), slightly outperformed the RFR method in one study [19]. In this last section, we put the RFR aside, after having used it as a valuable benchmark. Instead, we focus on developing a decision tree to identify simple mechanistically transparent and easy-to-understand models based mainly on GSE(*Φ,B*) and ABSOLV(GRP) [5].

As we checked specific molecules over the entire database *ad hoc* (or in the cases of outliers [5], discussed below), we found that the Consensus model was not always the best predictor. For example, we found that in cases of molecules with *Φ* < 1.66 (182 very rigid molecules), the Yalkowsky GSE(classic) was the best performing model, but just slightly so (*cf*. Table 3, and Fig. 2). Also, it is uncommon to find an example of a better simple predictor than the GSE(*Φ,B*) model for the space beyond *Φ* > 10.83 (342 very flexible molecules) (*cf*. Table 3, and Fig. 2). The span between *Φ* 1.66 and 10.83 (comprising 7144 entries– most of the database) revealed flip-flop in performance between GSE(*Φ,B*) and ABSOLV(GRP). Either the latter or the former was the best performer, *i.e*. an ‘exclusive or’ (XOR) behavior. How could one justify choosing the individual models over the Consensus model?

**Figure 1.**
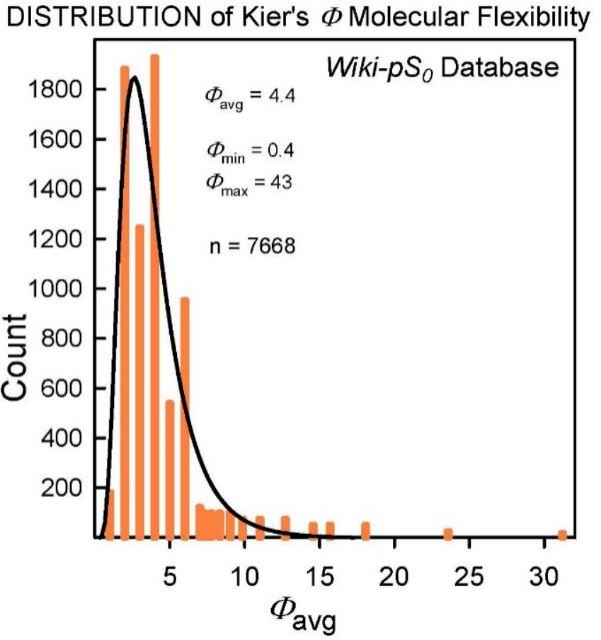
Distribution of the bin-average values of the molecular flexibility indices, *Φ*_avg_ (Table 3).

Insights to a possible answer to the above question may be revealed by further scrutinizing the performances of GSE(classic), ABSOLV(GRP) and GSE(*Φ,B*) models in the entire *Wiki-pS_0_* database. To start, all the entries in the database were sorted on *Φ* into 19 bins of near similar values of *Φ*. In each bin for each of the models, the RMSE value was calculated. Table 3 shows the bin errors distributions. Figure 1 illustrates the distribution of *Φ_avg_* counts in the bins.

**Figure 2.**
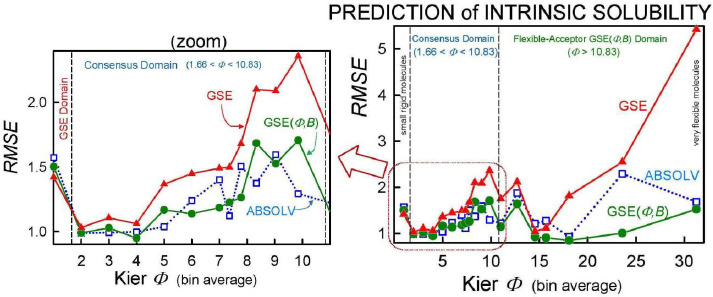
Distribution of root-mean-square (*RMSE*) values calculated for each of the bins in Table 3, as a function of Kier molecular flexibility index, *Φ*. For *Φ* < 1.66, the GSE slightly outperforms the other two models (as demarked by the vertical dashed line). For *Φ* > 10.83, the best performing simple model is GSE(*Φ,B*) – the Flexible-Acceptor model fit for flexible molecules. The zoom view on the left side of the drawing illustrates the GSE(*Φ,B*)-ABSOLV(GRP) flip-flop Consensus Domain region.

**Figure 3.**
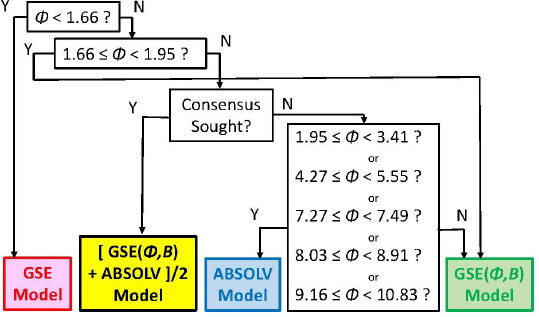
Decision Tree for the selection of the ‘best’ simple method for the prediction of intrinsic solubility. Either the Consensus model may be sought for 1.95 ≤ *Φ* < 10.83 by taking the average of the two best values, or the XOR Model may be invokes, by picking best of ABSOLV(GRP) and GSE(Φ,B) models.

Figure 2 compares the *RMSE* values distribution of the three models: GSE(classic) as red curves with triangle symbols, ABSOLV(GRP) as blue dotted curves with square symbols, and GSE(*Φ,B*) as green curves with circle symbols. A decision tree constructed based on the crossings in Figure 2 is shown in Figure 3.

Since the XOR Model in Figure 3 is dependent on the types of molecules within a particular bin of near constant flexibility, and since different databases may have molecules with differing properties in each range of *Φ* values, it may be prudent to select the Consensus model as the ‘best’ prediction. In our case of model training, the cross-over points may be sufficiently grounded to advance the XOR Model as the ‘best’ for the middle ‘Consensus Domain’ (*cf.* zoom view in Fig. 2). This is especially so, since this middle domain is comprised of many values of solubility, averaging over 1100 entries/bin for bins 2-7 (Table 3).

### Testing the decision tree model: Consensus vs. Exclusive Or (XOR) variants

The above Decision Tree considers the prediction potential from the view of the entire database. However, it is the performance of the predictions of *test set* molecules that really counts. So, we selected a slightly-increased set of 108 newly-approved FDA drugs to examine this last point.

- **Test Set 8** – Three additional log *S*_0_ values were added to Test Set 7 of newly FDA-approved drugs as more approvals were announced in 2022.

Table 4 summarizes the prediction metrics of the three simple models. Based on the properties of the newly-approved drugs in the test set, the ranking of the various methods is suggested. For of *r*^2^ and RMSE, the ranking of best performers follows: Consensus > GSE(*Φ,B*) > XOR Decision Tree > ABSOLV(GRP) > GSE(classic).

**Figure 4.**
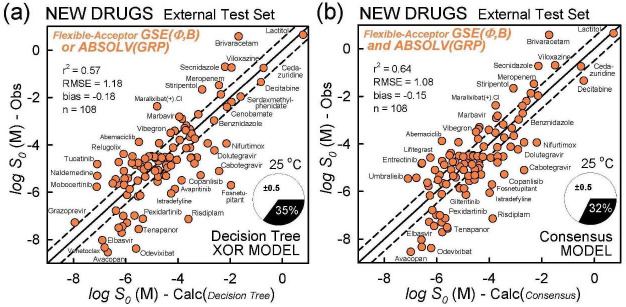
Prediction of newly-approved drugs (2016-2022). (a) XOR Decision Tree model predictions, compared to (b) Consensus model.

The model with the least *bias* is ABSOLV(GRP) and the model with the best *MPP* (more molecules within ±0.5 log of the correlation identity line) is the XOR Decision Tree (*cf*. Fig. 3). Figure 4 illustrates the differences between the XOR Decision Tree and Consensus models for the new drug predictions. The general scatter in the Consensus model is evidently less than that in the XOR model. Other minor differences are evident.

The summary in Table 4 may seem to be complicated, and different trends may be encountered for other test sets. In new test cases, the discussed simple models can be easily incorporated into an Excel spreadsheet (using the refined parameters in Tables 1 and 2) and compared for new cases.

### Another test example in model selection based on outliers

Oja *et al.* [5] responded to the Second solubility challenge [3,4] with three data-driven MLR models for predicting intrinsic aqueous solubility, which were mechanistically transparent and easily understandable. They discussed the challenges posed by outlier molecules.

Table 5 shows a different pattern of performance ranking compared to that in Table 4. ABSOLV(GRP) performed consistently in the first position for the four most flexible molecules. For the least flexible molecules, folic acid and cisapride, the XOR and GSE(*Φ,B*) looked promising. Our Consensus model lagged the others in this outliers example.

## Conclusion

Large, well-curated aqueous intrinsic solubility databases are available, with average interlaboratory reproducibility of less than 0.2 log unit. However, the distribution of drugs in the chemical space might not be uniform but may be sparsely populated in clusters [47]. Even a massive database might miss some clusters. Test compounds from such an under-represented portion of space may be poorly predicted, as the outliers in Table 5 could suggest. Also, the 32 ‘difficult-to-predict’ drugs in the Second Solubility Challenge [3,4] may be good examples of underpopulated cluster space, for which better representation is needed. It should also be noted that the 32 molecules are also ‘difficult-to-measure’ drugs.

The increasing use of ML/AI methods can lead to accurate predictions, as we have seen. However, these results may not readily suggest the steps to take to improve the properties of tested compounds. Our five studies in solubility prediction have attempted to match the performance of the Random Forest regression method, using relatively simple, mechanistically transparent, and easily applied models [5].

Increasingly, current trends in *in-silico* predictions of solubility use calculated input descriptors, which may be an advantage to explore properties of molecules yet to be synthesized. The risk may be that overall prediction approaches might be based on accumulated uncertainty, something that is often not emphasized [47]. The knowledge gained and predictive power applied to novel classes of test molecules can still be limited by the calculated descriptors.

Based on our latest findings, we recommend that both ABSOLV(GRP) and GSE(*Φ,B*) be calculated (*e.g*., by taking advantage of the refined parameters in Tables 1 and 2). For molecules with *Φ* < 11, the prudent choice is to pick the Consensus Model, the average of ABSOLV(GRP) and GSE(*Φ,B*). For more flexible molecules, GSE(*Φ,B*) is recommended.

## Figures and Tables

**Table 1.** ABSOLV(GRP) coefficients determined by PLS regression analysis of training set^a^ log *S*_0_^ABSOLV^ = *a*_0_ + *a*_1_*A* + *a*_2_*B* + *a*_3_*S*_*π*_ + *a*_4_*E* + *a*_5_*V* + *a*_6_*AB*

Group	*a*_0_	*a*_1_	*a*_2_	*a*_3_	*a*_4_	*a*_5_	*a*_6_	Pearson *r*^2^	RMSE	*n*
Acids	-0.30	0.26	1.07	0.04	-0.91	-1.80	0.43	0.65	1.17	1578
Bases	-0.39	-0.61	1.95	0.25	-1.67	-1.37	0.09	0.64	1.11	945
Zwitterions	1.52	-1.44	0.88	-0.23	-1.02	-1.08	0.38	0.71	0.88	641
Neutrals	-0.45	-0.18	1.73	0.10	-1.48	-1.36	0.26	0.63	1.12	4246
Big (MW > 800 Da)	-3.76	0.72	0.61	-0.03	-0.47	-0.38	-0.02	0.42	1.04	93
Quaternaries	0.53	-1.23	0.74	-0.04	-0.62	-0.49	0.09	0.66	0.94	39

^a^*a*_0_-*a*_6_ coefficients in Eq. (2). PLS open-source package from *https://cran.r-project.org/web/packages/pls*.

**Table 2.** Flexible-acceptor model, GSE(*Φ,B*)^a^ - log *S*_0_^GSE(Φ,B)^ = *c*_*0*_ + *c*_1_ clog *P* + *c*_2_ (*mp*-25)/100

Coefficients	Pearson *r*^2^	RMSE	number of bins
c_0_ = –4.456 + 6.049 e^–0.0817(*Φ,B*)^	0.93	0.41	20
*c*_1_ = –1.326 + 1.058 (1 - e^–0.1226(*Φ,B*)^ )	0.89	0.09	20
*c*_2_ = –0.941 + 0.0389(*Φ,B*)	0.55	0.27	20

^a^*b*_0_-*b*_7_ coefficients in Eq. (3) determined for the Wiki-p*S*_0_ database, using PLS regression: open-source package from *https://cran.r-project.org/web/packages/pls*.

**Table 3.** Model selection as a function of Kier flexibility, *Φ*

*Φ*_avg_ in Bin	Number of entries in bin	RMSE (GSE)	RMSE ABSOLV (GRP)	RMSE GSE(*Φ,B*)	‘Best’ model
1.0	182	1.42	1.57	1.50	GSE(classic)
2.0	1882	1.03	0.987	0.990	ABSOLV(GRP)
3.0	1245	1.11	0.99	1.03	ABSOLV(GRP)
4.0	1928	1.06	1.00	0.95	GSE(Φ,B)
5.0	539	1.37	1.04	1.17	ABSOLV(GRP)
6.0	953	1.45	1.24	1.14	GSE(Φ,B)
7.0	122	1.49	1.40	1.19	GSE(Φ,B)
7.4	100	1.50	1.12	1.23	ABSOLV(GRP)
7.8	100	1.68	1.50	1.26	GSE(Φ,B)
8.3	100	2.10	1.37	1.68	ABSOLV(GRP)
9.0	100	2.09	1.60	1.53	GSE(Φ,B)
9.9	75	2.36	1.29	1.71	ABSOLV(GRP)
11.0	75	1.75	1.22	1.15	GSE(Φ,B)
12.7	75	2.12	1.88	1.64	
14.6	50	1.04	1.22	0.93	
15.7	50	1.12	1.28	0.91	
18.1	50	1.82	0.93	0.85	
23.6	25	2.55	2.30	1.01	
31.2	17	5.42	1.69	1.53	

**Table 4.** Simple models for predicting solubility of newly-approved drugs^a^

Model	r^2^	RMSE	bias	MPP, %
Consensus	**0.64**	**1.08**	-0.15	32.4
GSE(*Φ,B*) Flexible-Acceptor	0.62	1.12	-0.32	33.3
XOR Decision Tree	0.57	1.18	-0.18	**35.2**
ABSOLV(GRP)	0.51	1.26	**0.02**	32.4
GSE(classic)	0.42	1.38	-0.36	31.5

^a^Test Set 8 with *n* = 108. The best metrics are highlighted in bold.

**Table 5.** Simple-model prediction of solubility of outliers in the Oja *et al.* [5] Study ^a^

Drug	*Φ*	log *S*_0_(Obs) [7]	Consensus [5]	Consensus [this work]	XOR Model	GSE(*Φ,B*) Flexible-Acceptor	ABSOLV (GRP)	GSE (classic)	Obs-Calc closest prediction
Folic Acid	6.6	-5.91 ± 0.17	-3.88	-2.51	**-4.07**	-2.97	-2.05	-1.71	-1.84
Cisapride	8.6	-6.78 ± 0.17	-4.21	-4.16	-2.97	**-4.24**	-4.07	-3.71	-2.54
Amiodarone	9.2	-10.40 ± 0.50	-7.86	-7.21	-4.38	-6.48	**-7.93**	-7.75	-2.47
Itraconazole	9.6	-8.71 ± 0.57	-7.27	-7.12	-7.93	-5.69	**-8.54**	-6.48	-0.17
Rifabutin	13.1	-3.99 ± 0.43	-6.81	-5.05	-8.54	-5.21	**-4.89**	-5.63	0.90
Cyclosporine A	31.5	-5.03 ± 0.16	-8.27	-4.49	-5.21	-4.38	**-4.59**	-4.03	-0.44

^a^Closest predictions are highlighted in bold

## Abbreviations and definitions

*S*_0_: intrinsic aqueous solubility (*i.e*. solubility of the uncharged form of the compound, in molarity units)
RMSE: correlation root-mean-square error: RMSE_*bias*_ = [ 1/(*n*-1) Σ_i_ (*y*_i_^obs^ - bias - *y*_i_^calc^)^2^ ]^1/2^, where *y*^obs^ or *y*^calc^ is the measured or calculated value of log *S*_0_, *n* = number of measurements of log *S*_0_. Note: some statistical programs define RMSE slightly differently: see discussion in Ref. [35]. ‘Validation’ type RMSE_val_ = [1/n Σ_i_ (*y*_i_^obs^ - *y*_i_^calc^)^2^]^1/2^
*r* ^2^: correlation coefficient of determination, *r*^2^_bias_ = 1 - Σ_i_(*y*_i_^obs^ - bias - *y*_i_^calc^)^2^ / Σ_i_(*y*_i_^obs^ - <*y*^obs^>)^2^, where *y* = log *S*_0_, and <*y*^obs^> is the mean value of measured log *S*_0_. Note: some statistical programs define *r*^2^ slightly differently. ‘Validation’ type [35] *r*^2^_val_ = 1 - Σ_i_(*y*_i_^obs^ - *y*_i_^calc^)^2^ / Σ_i_(*y*_i_^obs^ - <*y*^obs^>)^2^
bias: intercept (a) in the correlation fit: *y*^obs^ = *a* + *by*^calc^, where the slope factor *b* is fixed at unity.
SD: standard deviation: SD = [1/*n* Σ_i_(*y*_i_^obs^ - <y>)^2^]^1/2^, where *n* = number of measurements, <*y*> = mean value of log *S*_0_.
MPP: Measure of prediction performance [2]. It refers to the percent of ‘correct’ predictions, defined as the percentage of log residuals within 0.5 log unit of the identity line. MPP is represented as a pie chart in the correlation plots.

## Abraham solvation descriptors

A: H-bond total acidity (donor potential)
B: H-bond total basicity (acceptor potential)
S_π_: dipolarity/polarizability due to solute-solvent interactions between bond dipoles and induced dipoles
E: excess molar refraction (dm^3^ mol^-1^ / 10); which models dispersion force interaction arising from π- and n-electrons of the solute
V: McGowan molar volume (dm^3^ mol^-1^ / 100)
A·B: acid-base H-bonding product descriptor used in ABSOLV solubility prediction
